# Technical-Tactical Actions Used to Score in Taekwondo: An Analysis of Two Medalists in Two Olympic Championships

**DOI:** 10.3389/fpsyg.2019.02708

**Published:** 2019-12-10

**Authors:** Cristina Menescardi, Coral Falco, Concepción Ros, Verónica Morales-Sánchez, Antonio Hernández-Mendo

**Affiliations:** ^1^AFIPS Research Group, Department of Teaching of Musical, Visual and Corporal Expression, University of Valencia, Valencia, Spain; ^2^Department of Sport, Food and Natural Sciences, Western Norway University of Applied Sciences, Bergen, Norway; ^3^GIEPAFS (Grupo de Investigación en Educación Para una Actividad Física Saludable), Department of Motricity and Teaching in Physical Education, Universidad Católica de Valencia “San Vicente Mártir”, Valencia, Spain; ^4^Department of Social Psychology, Social Work, Anthropology and East Asian Studies, University of Málaga, Málaga, Spain

**Keywords:** techniques, tactics, polar coordinate, lag sequential, taekwondo, successful patterns

## Abstract

Research in taekwondo has traditionally focused on specific aspects athletes' overall technical and tactical skills, while ignoring other important issues such as identifying how successful athletes score points. The aim of the current study was to follow two medalists through two Olympic Championships (2012 and 2016) to discover the effective patterns associated with scoring in taekwondo using an observational methodology. An *ad-hoc* taekwondo observational tool was used to codify the actions performed by the athletes. An observational descriptive and multivariate analysis of 1,688 actions performed by the athletes was conducted. A lag sequential and polar coordinate analysis was performed that considered tactics, techniques and the kicking zone as factors related to score (one to four points). The results showed that one point was scored with direct attacks (DIAs) and actions to the chest where DIAs also occurred prior to scoring. After scoring one-point, opponents tended to gain points by kicking the opponent's head. Two points were scored with simultaneous spinning kicks. Cuts occurred prior to and after scoring while posterior counterattacks (PCAs) occurred after. Three points were scored by performing indirect attacks and PCAs to the head. Cuts, dodges, and linear actions preceded the three-point score while dodges, DIAs, and linear actions to the chest also occurred after the three point-actions. In conclusion, these two athletes not only mastered the whole tactics but also used specific strategies to score. That is, they anticipated the opponent's attack to score one point by kicking the opponent directly and performed spinning kicks from short distances when they perceived an opponent's cutting action to score two points. Finally, these athletes indirectly attacked the opponent when they dodged by kicking their head and counterattacked posteriorly when an opponent's cut was perceived to score three points. Thus, they used the most difficult tactics to achieve the highest score. It is suggested that coaches and psychologists train athletes in better decision-making by preparing them to not only prepare their own attacks but to systematically use the intended attacks by their opponents to score their own points in accordance with the successful patterns extracted in this study.

## Introduction

Scientific literature in taekwondo has shown a growing interest in performance indicators since it became an Olympic sport in the year 2000 (Cular et al., 2011). Research in taekwondo has traditionally focused on specific performance indicators related to the bout situation such as athletes' overall technical and tactical skills (Kazemi et al., 2006, 2009, 2010; Matsushigue et al., 2009; Cular et al., 2011; Santos et al., 2011; Tornello et al., 2014), which help to achieve a greater understanding of the dynamics of this sport.

Performance indicators related to score in this sport are tactics, techniques and the kicking zone (Falcó et al., 2012; Falco et al., 2014). Previous studies pointed out a specific competitive trend in terms of the tactics used by taekwondo college athletes (Falco et al., 2014). In this sense, the studied winning competitors performed more anticipatory counterattacks and less indirect attacks than non-winners. In addition, the use of direct, indirect attacks, and posterior counterattacks were the most performed action by winners and are suggested to increase the level of their tactical behavior. Olympic athletes tend to perform more attacks to score one point (Kazemi et al., 2006). Years later, the same research team found that 2004 Athens Olympics athletes more frequently used one-point attacks to score, followed by counterattacks and two-point attacks (Kazemi et al., 2009). They also concluded that the fighting style has become more dynamic as time passes. Unlike previous competitions, the 2008 Olympic competitors used more counterattacks to score, suggesting a more conservative style (Kazemi et al., 2010; Cular et al., 2011). Female Olympics competitors in 2012 used more attacks (direct and indirect) to score one and three points, respectively, while simultaneous counterattacks appeared to be the most performed to score two and four points (Menescardi, 2017), showing an association between a conservative style and one type of score (spinning techniques) and an aggressive style with other types of score (linear and circular techniques), showing that techniques were also related to score.

In terms of the techniques performed, it appears that circular techniques were the most frequently performed (Matsushigue et al., 2009) until London 2012, when the profile of the taekwondo techniques used changed from circular to other and more varied techniques, as well as increasing the number of kicks performed. This change can be seen as a consequence of the change in the point system, namely, the introduction of three points for kicks to the head and an extra point for a spinning kick with the inclusion of electronic chest protectors (Sledweski et al., 2015). In relation to techniques as well as scoring level, it is necessary to take into consideration the kicking zone as the location of the kicks determines their scoring (Falcó et al., 2012): one point for a valid attack on the chest protector, two points for a valid spinning kick to the chest protector, three points for a valid kick to the head with a linear or circular technique and four points for a valid spinning kick to the head (World Taekwondo Federation, 2012).

Purely descriptive studies of the mentioned variables have primarily been used to increase the scientific knowledge of taekwondo competitive situations, while pattern detection studies are still scarce. In this sense, the identification of successful patterns in taekwondo which allow athletes to score seems important due to the internal logic of the sport, since athletes win the bout if they score more than the opponent (Menescardi and Estevan, 2017). The ability to execute effective patterns through a combination of cognitive, perceptual, and motor skills is one of the most important aspects of an athlete's performance (Ali, 2011). Only five studies have analyzed patterns in taekwondo, two of which used lag sequential analysis (González-Prado et al., 2015; Menescardi and Estevan, 2017) while the last three (López-López et al., 2015; Menescardi et al., 2016, 2019) used polar coordinate analysis.

A previous study that used lag sequential analyses showed that the offensive actions performed in 48 bouts from six World Cups (2000–2008) act in favor of further effective actions, while defensive actions act as inhibitors. In addition, the advantage in the scoreboard acts as an inhibitor of offensive actions and winning athletes then tend to use defensive actions (González-Prado et al., 2015). Another study showed an interchangeable pattern of attacking and counterattacking actions in which attacking actions were followed by simultaneous counterattacks or dodges, while counterattacks (anticipatory, simultaneous, or posterior) were followed by attacks (Menescardi and Estevan, 2017). Menescardi et al. (2016) found similar results by using polar coordinate analysis. López-López et al. (2015) found a relationship between anticipatory counterattacks with circular kicks performed with the left leg to score one point in Olympic males (London 2012) due to anticipated actions surprised the opponent. In addition, anticipatory counterattacks and spinning kicks were related to effective two-point actions, while three-point actions were related to circular techniques, the most frequently performed in competitions (Matsushigue et al., 2009). Finally, Menescardi et al. (2019) found different patterns between male and females in one-point actions: female scored one point after cut, direct attacks with circular techniques to the chest, while males scored after spinning anticipate counterattack and dodges. Females scored two points after direct attacks while males used simultaneous counterattacks to the head. Both, males and females, scored three points after cuts and posterior counterattacks. Males scored four points after blocks and simultaneous counterattacks.

The scientific literature in this sport has focused on the technical-tactical aspects of all competitors and has ignored other important issues such as identifying the behavior of talented or successful athletes. The analysis of a specific player's performances has been carried out in other sports such as soccer by analyzing the behavior of Xavier Hernández (Maneiro et al., 2019), Lionel Messi and Cristiano Ronaldo (Castañer et al., 2016, 2017). Similar studies have still not been performed in the field of taekwondo. Consequently and in view of the data presented, a thorough study using an effective analysis technique such as polar coordinate and lag sequential analysis is justified. To analyze the decisions taken in sport, it is necessary to analyze the different actions that occur during a bout and both lag sequential and polar coordinate analysis are suitable techniques for identifying and helping to understand these actions (Morillo et al., 2017; Prudente et al., 2017). The first technique analyzes which actions occurred before and after a behavior of interest (i.e., focal behavior) and other behaviors (i.e., conditional behaviors; Castañer et al., 2017) with the number of lags related to the internal logic of the sport and considered relevant by researchers. The second technique reduces the volume of the data analyzed to highlight significant relationships between focal and conditioned behaviors in an easy-to-interpret vector map showing what happens right before and after the behavior of interest (Morillo et al., 2017). These techniques are complementary (Castañer et al., 2017; Tarragó et al., 2017) and with important methodological endorsements in areas of social sciences such as those pertaining to sport (Hernández-Mendo and Anguera, 2002; Perea et al., 2012; López-López et al., 2015; Zourbanos et al., 2015; Castañer et al., 2016, 2017; Arias-Pujol and Anguera, 2017; Morillo et al., 2017; Tarragó et al., 2017; Maneiro and Amatria, 2018; Pérez-Tejera et al., 2018; Maneiro et al., 2019).

Given the scarcity of studies analyzing the behavior of elite taekwondo athletes, the aim of the present study was to follow two medalists through two Olympic Championships (London 2012 and Rio de Janeiro 2016) and conduct a lag sequential and polar coordinate analysis to discover effective patterns in relation to tactics, techniques and the kicking zone associated with different scores in taekwondo using an observational methodology.

## Materials and Methods

### Methodology and Design

This study used an observational methodology, which has been proven to be one of the most suitable research methods for studying the spontaneous interactions between athletes (Maneiro and Amatria, 2018). The design of the present study is I/S/M, where I refers to ideographic (focusing on two athletes), S refers to intersessional follow-up (recording of eight bouts) and intrasessional follow-up (continuous recording of specific moves), and M refers to multidimensional (analysis of multiple criteria or levels of response, using a purpose-designed observation instrument).

### Participants and Sample

A total of 1,688 actions performed by two athletes during two Olympic tournaments (2012 London and 2016 Rio de Janeiro) were coded from public television broadcasts and analyzed. The athletes studied were Jade Jones and Lee Dae-hoon (57 and 68 kg, respectively), who were medalists in both tournaments and the only two medalists still currently active. Olympic medalists competed on four bouts (i.e., preliminary, quarterfinal, semifinal, and final round) in each tournament carried out on the same day (for each weight category) from morning to evening. The criterion for action inclusion was the clear observability of each action; when the action was not clear or incomplete, it was not codified. This research was carried out in accordance with the Declaration of Helsinki. Since the analyzed videos in which the public behavior can be observed are in the public domain, it was not necessary to acquire informed consent from the athletes concerned (American Psychological Association, 2002). The study protocol was approved by the Human Research Ethics Committee of the University of Valencia.

### Materials

To code the athletes' behavior, the taekwondo observational tool, validated by Menescardi et al. (2017) was used (Table 1), which contains four exhaustive category systems (criteria) and 19 mutually exclusive categories to characterize the taekwondo actions distributed within each criterion. HOISAN software (Hernández-Mendo et al., 2012) was used to codify the data and conduct the lag sequential and polar coordinate analysis.

**Table 1:** Categories, codes, and categorical core of the observational tool used.

**Criteria**	**Categories**	**Code**	**Categorical core or description**
Tactics	Direct attack	DIA	Offensive action with the objective of scoring, ending with an impact on the opponent but without previous movement
	Indirect attack	INA	Offensive action in order to score, ending with an impact on the opponent and with previous movement such as a step, skip, opening, guard change, kicking trajectory modification, etc.
	Anticipated counterattack	ACA	Action that starts during the opponent's attack with the purpose of scoring. The athlete kicks the attacker during the preparatory phase (guard) and/or initial phase (when the opponent's knee is being raised)
	Simultaneous counterattack	SCA	Action that starts at the same time as the opponent's attack and has a scoring purpose. The athlete kicks at the same time as the opponent. Thus, the counter attacker kicks at the end of the attacker's initial phase (leg raised) or during the impact momentum (impact phase) of the attacker's kick
	Posterior counterattack	PCA	Action that begins after the opponent's attack (during the descending phase, or when attacker's leg touches the ground) with a scoring purpose. Athletes kick at the same time. This action (sometimes) includes a previous backward displacement to dodge the opponent's attack
	Opening	OPE	Movement to control the distance with the opponent or bridge the gap between both competitors
	Block	BLO	Defensive actions to avoid the impact of a kick by placing one arm or leg between the protector and the leg of the opponent. This does not have a scoring objective
	Dodge	DOD	Defensive actions to avoid the impact of a kick by placing one arm or leg between the protector and the leg of the opponent. This does not have a scoring objective
	Cut	CUT	Defensive forward movement to avoid being beaten by a close opponent, and to prevent the attacking action from being completed. This does not have a scoring objective
Techniques	Linear	LIN	The kicking leg is directed toward the front of the opponent's body with a pushing motion in an attempt to kick the opponent with the sole of the foot
	Circular	CIR	The kicking leg is directed toward the opponent's side, with a circular movement in an attempt to kick the opponent with the instep
	Spin	GIR	Action performed with a previous rotation, at least 180° from the initial position, before kicking the opponent
Kicking zone	Trunk	PET	Kick to permitted areas of the trunk
	Head	CAB	Kick to permitted areas of the head
Score	0 points	SC0	Action does not impact on the permitted areas, or impacts in these areas but not with enough force to score
	1 point	SC1	Score obtained by a valid action performed to the trunk with a linear or circular technique
	2 points	SC2	Score obtained by a valid action performed to the trunk using a spin beforehand (in 2012)
	3 points	SC3	Score obtained by a valid action performed to the head with a linear or circular technique or spinning technique to the chest (in 2016)
	4 points	SC4	Score obtained by a valid action performed to the head using a spin beforehand

### Procedure

The actions were analyzed by different observers previously trained following the procedure stated by Anguera (2003). Six observers divided into two groups (Groups A and B) were involved in the reliability analysis of the data and analyzed six combat bouts to obtain the inter-observer reliability. Intra-observer reliability was analyzed by evaluating one observer's codification (six combat bouts) twice. Cohen's kappa (κ) was used to calculate intra- and inter-observer reliability, with κ values above 0.85 indicating almost perfect conformity (Landis and Koch, 1977; López-López et al., 2015).

### Statistical Analysis

Lag sequential analysis followed by polar coordinate analysis of the technical-tactical elements that influence the score were performed using HOISAN software. Previous studies have applied both statistical analysis due to its complementarity in observational studies in the field of sport (Castañer et al., 2017; Tarragó et al., 2017). This software integrates the analysis of the prospective and retrospective lag sequential analysis, allowing researchers to detect when technical-tactical elements prior to the score are revealed as preparatory to the occurrence of each score (retrospective) while the prospective perspective is based on considering those elements as subsequent to the score (*Z* > 1.96; *p* < 0.05). The number of negative (−1 and −2) and positive lags (+1 and +2) were used for known behavior associations between focal and conditioned behaviors (that is, technical-tactical elements and score), in line with Anguera et al. (2018). Polar coordinate analysis combines adjusted residuals from lag sequential analysis and the Zsum statistic (Anguera et al., 2018). To do that, adjusted residuals (AR) derived from sequential analysis (Z scores) were used to calculate Zsum statistics (Zsum = Pz/√n) (Cochran, 1954). AR are computed by comparing observed with expected probabilities (chance occurrences). The relationship between the focal behavior and the conditional behaviors in polar coordinate analysis is estimated using the angle of the resulting vector, while the strength is estimated using the vector radius as √(ZsumProspective^2^+ZsumRetrospective^2^) (Arias-Pujol and Anguera, 2017). The resulting polar coordinate maps show the associations between the focal and conditional behaviors analyzed depending on the angle and the quadrant in which behaviors appeared. The angle (θ) is determined by the sine arc of Y/Radio, giving rise to four relationships depending on the quadrant in which the vector is located:

- Quadrant I (0–90°). Indicates that the focal and conditional behaviors are mutually activated in both perspectives, that is, the conditioned behaviors occur before and after the focal behavior (+, +).
- Quadrant II (90–180°). Indicates that the focal behavior inhibits the conditional behaviors but is also activated by them, that is, the conditioned behavior precedes but does not follow the focal behavior (+, –).
- Quadrant III (180–270°). Indicates that the focal and conditional behaviors are mutually inhibited, that is, the conditioned behavior neither precedes nor follows the focal behavior (–, –).
- Quadrant IV (270–360°). Indicates that the focal behavior activates the conditional behaviors but is also inhibited by them, that is, the conditioned behavior does not precede but follows the focal behavior (–, +).

Despite the fact that all relationships appear in the vector representation of the polar coordinates map, only those with a module or radium length (r) of the vector > 1.96 are considered significant (*p* < 0.05) and included in the results. The relations between the behaviors were represented on a vector map using MATLAB software (Perea et al., 2012). Taking into account the goal of the study, a total of four polar coordinates analyses were conducted and the score criteria included four options: one point, two points, three points, and four points from the pooled data, for each competitor and each tournament.

## Results

### Results From the Pooled Data

**Figure 1:**
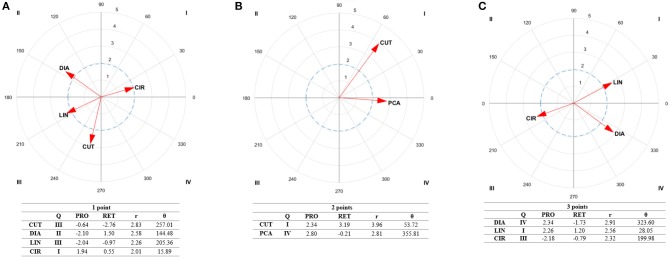
Representation of behavioral maps for 1-point **(A)**, 2-point **(B)**, and 3-point **(C)** effective actions as focal behavior. The behavioral map was represented divided into four quadrants, with each of the conditioned categories as vectors in the axis X/Y and their respective coordinates Z_sum_ prospective (X) and Z_sum_ retrospective (Y). Significate vectors (Radium > 1.96), Zsum values, prospective (pros.) and retrospective (retr.) perspective, quadrants (Q), radium length (r), and vector angle (θ) are represented. CUT, Cuts; DIA, Direct Attack; PCA, Posterior Counterattack; LIN, linear technique; CIR, Circular technique.

A lag sequential (Table 2) and polar coordinate analysis were performed to investigate factors that explain how successful athletes achieve their score (one to three points) and win their bouts. No significant associations were found with four-point actions, as elite athletes performed none during the bouts analyzed. The results of lag sequential analysis showed a relationship between one point-actions and direct attacks (*Z*_−2_ = 2.51; *Z*_0_ = 2.46) and actions to the chest (*Z*_0_ = 8.01) and to the head (*Z*_+1_ = 2.46). Relationships between two point-actions were found with cuts (*Z*_−2_ = 4.76; *Z*_+2_ = 3.52), posterior counterattacks (*Z*_+1_ = 4.18) and spinning techniques (*Z*_0_ = 4.90). Relationships between three point-actions were found with dodges (*Z*_−2_ = 2.76), indirect attacks (*Z*_0_ = 2.11) and posterior counterattacks (*Z*_0_ = 2.32) to the head (*Z*_0_ = 7.68), linear actions (*Z*_+1_ = 2.03) and actions to the chest (*Z*_+__1_ = 2.33). The results of polar coordinate analysis showed significant associations (*r* > 1.96; *p* < 0.05) between the different types of score and actions used to score (Figure 1). That is, direct attacks occurred prior to one-point actions (QII, *r* = 2.58) and after three-point actions (QIV, *r* = 2.91). Circular techniques occurred prior to one-point actions (QI, *r* = 2.01). Cuts were performed prior to and after two-point actions (QI, *r* = 3.96). Linear techniques occurred prior to and after three-point-actions (QI, *r* = 2.56). Posterior counterattacks occurred after two-point actions (QIV, *r* = 2.81). No significant associations were found with the other variables studied (Table 2). Supplemental Material with a graphical representation of behavioral patterns extracted from lag sequential were included with their corresponding screenshot to give readers additional information about the results obtained.

**Table 2:** Lag sequential analysis of two lags (−2, +2) results.

	**1 point**					**2 points**					**3 points**				
**Lags:**	**−2**	**−1**	**0**	**+1**	**+2**	**−2**	**−1**	**0**	**+1**	**+2**	**−2**	**−1**	**0**	**+1**	**+2**
BLO	−1.57	−0.20	0.00	0.38	0.88	−0.21	−0.21	0.00	−0.27	−0.18	1.66	0.26	0.00	−0.32	−0.85
DOD	−2.57	0.97	0.00	1.02	0.20	−0.53	−0.45	0.00	−0.64	−0.48	**2.76**	−0.87	0.00	−0.86	−0.07
CUT	−2.90	−1.00	0.00	−0.24	−0.66	**4.76**	−0.25	0.00	−0.21	**3.52**	1.66	1.08	0.00	0.29	−0.31
OPE	0.81	−0.44	0.61	−0.07	−0.98	−0.34	−0.45	−0.12	−0.33	−0.43	−0.73	0.56	−0.59	0.16	1.12
DIA	**2.51**	−0.39	**2.46**	−1.67	−1.30	−0.61	1.67	−0.88	−0.47	−0.62	−2.39	−0.05	−2.26	1.82	1.49
INA	−0.36	0.21	−1.96	0.20	0.61	−0.31	−0.36	−0.44	−0.45	−0.32	0.45	−0.12	**2.11**	−0.09	−0.53
ACA	0.61	−0.20	1.82	0.62	0.62	−0.12	−0.21	−0.35	−0.12	−0.12	−0.59	0.26	−1.76	−0.60	−0.59
SCA	1.21	0.42	−1.50	−0.07	1.17	−0.50	−0.38	1.84	−0.33	−0.40	−1.09	−0.33	1.03	0.16	−1.08
PCA	0.88	0.63	−2.22	0.11	1.09	−0.17	−0.12	−0.24	**4.18**	−0.22	−0.84	−0.61	**2.32**	−1.22	−1.04
LIN	0.33	−1.70	1.05	−1.50	−1.39	0.00	−0.85	−1.01	−0.84	0.00	−0.33	**2.03**	−0.80	1.80	1.39
CIR	−0.55	1.33	0.10	1.71	1.03	0.00	0.93	−0.93	0.92	0.00	0.55	−1.67	0.14	−2.05	−1.03
GIR	0.55	0.88	−2.91	−0.57	0.86	0.00	−0.21	**4.90**	−0.22	0.00	−0.55	−0.83	1.66	0.65	−0.86
PET	0.54	0.74	**8.01**	−2.46	1.70	0.00	0.21	−1.78	0.55	0.00	−0.54	−0.83	−7.68	**2.33**	−1.70
CAB	−0.54	−0.74	−8.01	**2.46**	−1.70	0.00	−0.21	1.78	−0.55	0.00	0.54	0.83	**7.68**	−2.33	1.70

*Bold front means significate vectors (Z > 1.96). Z values of retrospective lags (−2, −1), focal behavior (0) and prospective lags (+1, +2) are represented. (–) behaviors have an inhibitory effect while positive behaviors have an excitatory (+) effect. BLO, blocks; DOD, dodges; CUT, cuts; OPE, opening; DIA, direct attack; INA, indirect attack; ACA, anticipatory counterattack; SCA, simultaneous counterattack; PCA, posterior counterattack; LIN, linear technique; CIR, circular technique; GIR, spinning technique; PET, action to the chest protector; CAB, action to the head*.

### Competitor's Styles

**Figure 2:**
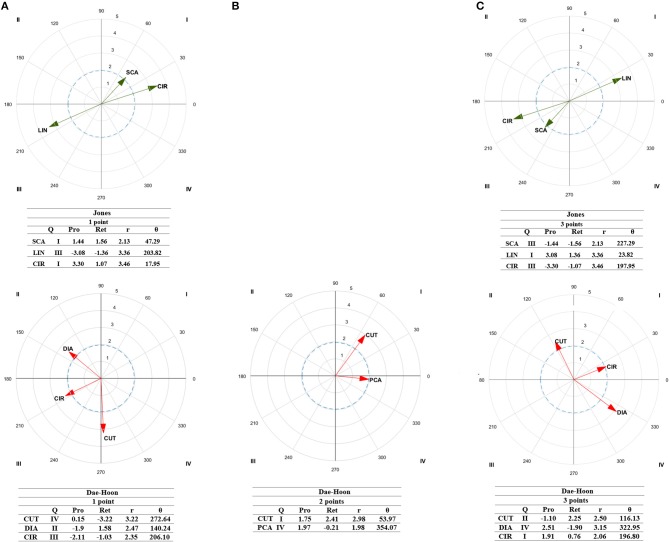
Representation of behavioral maps for 1-point **(A)**, 2-point **(B)**, and 3-point **(C)** effective actions as focal behavior for Jones (green) and Dae-Hoon (red). The behavioral map was represented divided into four quadrants, with each of the conditioned categories as vectors in the axis X/Y and their respective coordinates Z_sum_ prospective (X) and Z_sum_ retrospective (Y). Significate vectors (Radium > 1.96), Zsum values, prospective (pros.) and retrospective (retr.) perspectives, quadrants (Q), radium length (r), and vector angle (θ) are represented. DOD, Dodges; CUT, Cuts; DIA, Direct Attack; PCA, Posterior Counterattack; LIN, linear technique; CIR, Circular technique.

Considering lag sequential (Table 3), to score one point, Jones scored with actions to the chest (*Z*_0_ = 6.27), while circular techniques (*Z*_+1_ = 3.39) and actions to the head (*Z*_+1_ = 2.10) occurred posteriorly. No significant associations were found with two-point actions, as she performed none during the bouts analyzed. To score three points, she performed dodges (*Z*_−2_ = 2.68), and scored with indirect attacks to the head (*Z*_0_ = 2.74 and 6.27, respectively) while linear actions to the chest occurred after scoring (*Z*_+1_ = 2.83 and 2.10, respectively). Polar coordinate analysis (Figure 2) showed that to score one point she performed simultaneous counterattacks prior to and after scoring (QI, *r* = 2.13) with circular techniques (QI, *r* = 3.46). Prior to and after scoring three-points she used also linear techniques (QI, *r* = 3.36).

**Table 3:** Lag sequential analysis of Jones and Dae-Hoon of two lags (−2, +2) results.

	**1 point**					**2 points**					**3 points**				
**Lags:**	**−2**	**−1**	**0**	**+1**	**+2**	**−2**	**−1**	**0**	**+1**	**+2**	**−2**	**−1**	**0**	**+1**	**+2**
	**Jones**														
BLO	−1.42	−0.09	0.00	−0.15	0.99	–	–	–	–	–	1.42	0.09	0.00	0.15	−0.99
DOD	−2.68	1.04	0.00	1.15	−0.12	–	–	–	–	–	**2.68**	−1.04	0.00	−1.15	0.12
CUT	−1.54	−0.09	0.00	−1.56	−0.57	–	–	–	–	–	1.54	0.09	0.00	1.56	0.57
OPE	0.24	−0.17	0.66	−0.54	−0.70	–	–	–	–	–	−0.24	0.17	−0.66	0.54	0.70
DIA	1.95	−1.30	1.74	−0.84	−1.24	–	–	–	–	–	−1.95	1.30	−1.74	0.84	1.24
INA	1.18	−0.87	−2.74	−0.28	−0.06	–	–	–	–	–	−1.18	0.87	**2.74**	0.28	0.06
ACA	0.67	−0.09	1.54	0.00	0.00	–	–	–	–	–	−0.67	0.09	−1.54	0.00	0.00
SCA	0.80	1.41	−0.28	0.74	1.30	–	–	–	–	–	−0.80	−1.41	0.28	−0.74	−1.30
PCA	0.00	0.68	−1.56	0.66	0.99	–	–	–	–	–	−0.00	−0.68	1.56	−0.66	−0.99
LIN	−0.14	−1.78	0.87	−2.83	−1.52	–	–	–	–	–	0.14	1.78	−0.87	**2.83**	1.52
CIR	0.00	1.51	−0.40	**3.39**	1.27	–	–	–	–	–	−0.00	−1.51	0.40	−3.39	−1.27
GIR	0.40	0.74	−1.56	−1.48	0.70	–	–	–	–	–	−0.40	−0.74	1.56	1.48	−0.70
PET	1.07	1.40	**6.27**	−2.10	1.48	–	–	–	–	–	−1.07	−1.40	−6.27	**2.10**	−1.48
CAB	−1.07	−1.40	−6.27	**2.10**	−1.48	–	–	–	–	–	1.07	1.40	**6.27**	−2.10	1.48
	**Dae–Hoon**														
BLO	0.00	0.00	0.00	0.83	0.00	0.00	0.00	0.00	−0.28	0.00	0.00	0.00	0.00	−0.75	0.00
DOD	−0.74	0.24	0.00	0.16	0.92	−0.54	−0.46	0.00	−0.68	−0.38	1.06	−0.04	0.00	0.14	−0.81
CUT	−2.75	−1.80	0.00	0.83	−0.62	**3.60**	−0.19	0.00	−0.28	**2.76**	1.19	**1.99**	0.00	−0.75	−0.81
OPE	0.98	−0.39	0.00	0.83	−0.62	−0.36	−0.35	0.00	−0.28	−0.38	−0.88	0.57	0.00	−0.75	0.87
DIA	1.64	0.59	1.77	−1.66	−1.02	−0.60	1.30	−0.89	−0.52	−0.83	−1.47	−1.21	−1.47	**1.99**	1.56
INA	−1.96	1.22	1.01	1.03	0.92	−0.36	−0.41	−0.34	−0.35	−0.38	**2.28**	−1.10	−0.91	−0.93	−0.81
ACA	0.00	0.00	1.01	0.57	0.51	0.00	0.00	−0.34	−0.19	−0.21	0.00	0.00	−0.91	−0.52	−0.45
SCA	1.16	−0.91	−2.08	−1.80	0.51	−0.42	−0.46	1.69	−0.19	−0.21	−1.03	1.17	1.45	**1.99**	−0.45
PCA	0.79	0.00	−1.87	−0.39	0.51	−0.29	0.00	−0.34	**3.00**	−0.21	−0.70	0.00	**2.13**	−0.93	−0.45
LIN	0.89	0.11	1.04	1.48	0.85	0.00	−0.63	−0.67	−0.57	0.00	−0.89	0.25	−0.80	−1.27	−0.85
CIR	−1.14	−0.32	0.39	−1.97	−1.01	0.00	0.70	−1.34	0.76	0.00	1.14	−0.06	0.19	1.69	1.01
GIR	0.35	0.48	−2.65	0.67	0.46	0.00	−0.22	**3.81**	−0.26	0.00	−0.35	−0.41	1.10	−0.57	−0.46
PET	−0.53	−0.48	**4.96**	−1.30	0.00	0.00	0.22	**2.03**	0.50	0.00	0.53	0.41	−4.34	1.11	0.00
CAB	0.53	0.48	−4.96	1.30	0.00	0.00	−0.22	−2.03	−0.50	0.00	−0.53	−0.41	**4.34**	−1.11	0.00

*Bold front means significate vectors (Z > 1.96). Z values of retrospective lags (−2, −1), focal behavior (0) and prospective lags (+1, +2) are represented. (–) behaviors have an inhibitory effect while positive behaviors have an excitatory (+) effect. BLO, blocks; DOD, dodges; CUT, cuts; OPE, opening; DIA, direct attack; INA, indirect attack; ACA, anticipatory counterattack; SCA, simultaneous counterattack; PCA, posterior counterattack; LIN, linear technique; CIR, circular technique; GIR, spinning technique; PET, action to the chest protector; CAB, action to the head*.

Regarding Dae-Hoon's style, lag sequential analysis (Table 3) showed that he used actions to the chest (*Z*_0_ = 4.96) to score one point. Also, a relationship was found with cuts and two-points (*Z*_−2_ = 3.60) and spinning actions to score (*Z*_0_ = 3.81) followed by posterior counterattacks (*Z*_+1_ = 3.00) and cuts (*Z*_+2_ = 2.76). Regarding, three-points actions, a relationship was found between indirect attacks (*Z*_−2_ = 2.28) and cuts (*Z*_−1_ = 1.99) prior to scoring while he used posterior counterattacks to the head to score (*Z*_0_ = 2.13 and 4.34, respectively) and direct attacks (*Z*_+1_ = 1.99) in addition to simultaneous counterattacks (*Z*_+1_ = 1.99) occurred after that. Polar coordinate analysis (Figure 2) indicates he used direct attacks prior to scoring one-point (QII, *r* = 2.47) while cuts occurred after one point score (QIV, *r* = 3.22). A relationship was found between cuts and two-point actions (QI, *r* = 2.98) which are followed by posterior counterattacks (QIV, *r* = 1.98). Regarding, three-point actions, cuts occurred prior to scoring (QII, *r* = 2.50) while he used direct attacks (QIV, *r* = 3.15) after scores. Circular techniques were performed prior to and after scoring (QI, *r* = 2.06). Supplemental Videos were included with patterns extracted from both competitors.

### Tournament's Styles

Regarding lag sequential (Table 4), in London 2012, direct attacks (*Z*_−2_ = 2.03) occurred prior to scoring one point throughout kicks to the chest (*Z*_0_ = 6.56). To score two points, cuts occurred prior to scoring (*Z*_−2_ = 6.40), while posterior counterattacks (*Z*_+1_ = 3.16) and cuts (*Z*_+2_ = 4.30) occurred after scoring. Two-point actions were scored with simultaneous counterattacks (*Z*_0_ = 1.97) and spinning techniques (*Z*_0_ = 6.56) to the chest (*Z*_0_ = 2.12). To score three points, athletes used dodges prior to scoring (*Z*_−2_ = 2.32) while actions were scored with kicks to the head (*Z*_0_ = 6.05). After scoring, indirect attacks (*Z*_+1_ = 2.08) and linear techniques (*Z*_+2_ = 1.98) were performed. In Rio de Janeiro 2016, athletes scored one-point with direct attacks (*Z*_0_ = 2.53) and linear techniques (*Z*_0_ = 2.22) to the chest (*Z*_0_ = 4.83). Actions to the head (*Z*_+1_ = 2.10) and indirect attacks (*Z*_+1_ = 2.00) occurred after scoring. No two-point actions were significant. In addition, three-point actions were scored with kicks to the head (*Z*_0_ = 4.83) and indirect attacks (*Z*_0_ = 2.07). After scoring, actions to the chest occurred (*Z*_+1_ = 2.10).

**Table 4:** Lag sequential analysis of 2012 and 2016 Olympic Tournaments.

	**1 point**					**2 points**					**3 points**				
**Lags:**	**−2**	**−1**	**0**	**+1**	**+2**	**−2**	**−1**	**0**	**+1**	**+2**	**−2**	**−1**	**0**	**+1**	**+2**
	**London 2012**														
BLO	−1.12	0.50	0.00	0.86	0.52	−0.23	−0.16	0.00	−0.28	−0.17	1.27	−0.46	0.00	−0.79	−0.48
DOD	−2.04	1.04	0.00	1.95	−0.31	−0.42	−0.33	0.00	−0.63	−0.52	**2.32**	−0.96	0.00	−1.80	0.54
CUT	−2.06	0.50	0.00	−1.17	−1.03	**6.40**	−0.16	0.00	−0.22	**4.30**	−0.46	−0.46	0.00	1.32	−0.69
OPE	0.19	−0.44	0.00	−0.74	−1.03	−0.42	−0.50	0.00	−0.45	−0.56	−0.03	0.66	0.00	0.96	1.32
DIA	**2.03**	−0.96	1.07	−0.09	−0.31	−0.65	1.78	−0.86	−0.37	−0.52	−1.87	0.28	−0.78	0.24	0.54
INA	−0.29	0.38	−0.74	−1.80	1.09	−0.33	−0.46	−0.45	−0.45	−0.35	0.44	−0.22	0.96	**2.08**	−1.00
ACA	0.50	−0.63	1.38	0.00	0.52	−0.16	−0.28	−0.45	0.00	−0.17	−0.46	0.78	−1.28	0.00	−0.48
SCA	0.38	0.19	−1.28	0.86	0.93	−0.46	−0.42	**1.97**	−0.28	−0.30	−0.22	−0.03	0.54	−0.79	−0.86
PCA	0.71	0.50	−1.17	−0.35	0.75	−0.23	−0.16	−0.22	**3.16**	−0.24	−0.66	−0.46	1.32	−0.93	−0.69
LIN	0.16	−1.69	−0.37	−1.33	−1.98	0.00	−0.78	−0.90	−1.02	0.00	−0.16	**2.10**	0.75	1.86	**1.98**
CIR	−0.38	1.28	1.00	1.33	1.58	0.00	0.88	−1.09	1.02	0.00	0.38	−1.71	−0.62	−1.86	−1.58
GIR	0.47	0.79	−2.12	0.00	0.70	0.00	−0.25	**6.56**	0.00	0.00	−0.47	−0.73	−0.45	0.00	−0.70
PET	−0.33	−0.55	**6.56**	−1.51	0.00	0.00	0.17	**2.12**	0.50	0.00	0.33	0.51	−6.05	1.36	0.00
CAB	0.33	0.55	−6.56	1.51	0.00	0.00	−0.17	−2.12	−0.50	0.00	−0.33	−0.51	**6.05**	−1.36	0.00
	**Rio de Janeiro 2016**														
BLO	−1.30	−0.30	0.00	−0.30	0.76	–	–	–	–	–	1.30	0.30	0.00	0.30	−0.76
DOD	−1.31	1.01	0.00	−0.33	0.48	–	–	–	–	–	1.31	−1.01	0.00	0.33	−0.48
CUT	−1.87	−0.99	0.00	0.83	0.09	–	–	–	–	–	1.87	0.99	0.00	−0.83	−0.09
OPE	0.80	−0.44	0.81	0.00	−1.37	–	–	–	–	–	−0.80	0.44	−0.81	0.00	1.37
DIA	1.48	0.49	**2.53**	−1.52	−1.18	–	–	–	–	–	−1.48	−0.49	−2.53	1.52	1.18
INA	−0.36	−1.25	−2.07	**2.00**	−0.44	–	–	–	–	–	0.36	1.25	**2.07**	−2.00	0.44
ACA	0.00	0.00	0.81	0.83	0.00	–	–	–	–	–	0.00	0.00	−0.81	−0.83	0.00
SCA	1.48	0.25	−0.76	−0.44	1.10	–	–	–	–	–	−1.48	−0.25	0.76	0.44	−1.10
PCA	0.00	0.00	−1.84	0.00	0.76	–	–	–	–	–	0.00	0.00	1.84	0.00	−0.76
LIN	0.40	−0.35	**2.22**	−1.14	−0.05	–	–	–	–	–	−0.40	0.35	−2.22	1.14	0.05
CIR	−0.40	0.35	−1.34	1.27	0.05	–	–	–	–	–	0.40	−0.35	1.34	−1.27	−0.05
GIR	0.00	0.00	−1.84	−0.34	0.00	–	–	–	–	–	0.00	0.00	1.84	0.34	0.00
PET	0.37	1.32	**4.83**	−2.10	1.35	–	–	–	–	–	−0.37	−1.32	−4.83	**2.10**	−1.35
CAB	−0.37	−1.32	−4.83	**2.10**	−1.35	–	–	–	–	–	0.37	1.32	**4.83**	−2.10	1.35

*Bold front means significate vectors (Z > 1.96). Z values of retrospective lags (−2, −1), focal behavior (0) and prospective lags (+1, +2) are represented. (–) behaviors have an inhibitory effect while positive behaviors have an excitatory (+) effect. BLO, blocks; DOD, dodges; CUT, cuts; OPE, opening; DIA, direct attack; INA, indirect attack; ACA, anticipatory counterattack; SCA, simultaneous counterattack; PCA, posterior counterattack, LIN, linear technique; CIR, circular technique; GIR, spinning technique; PET, action to the chest protector; CAB, action to the head*.

**Figure 3:**
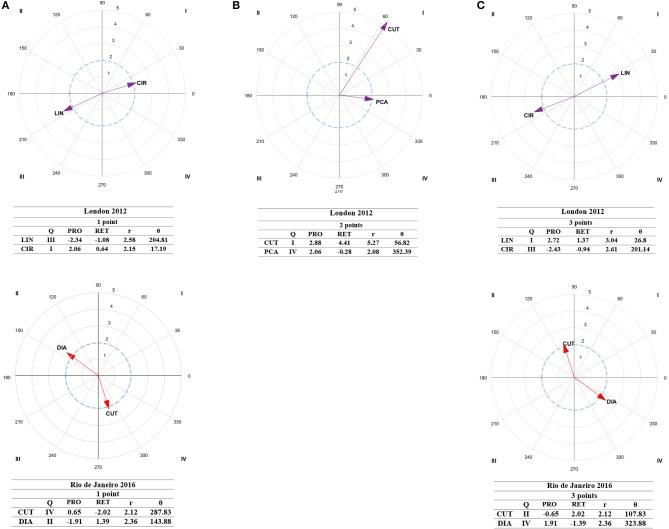
Representation of behavioral maps for 1-point **(A)**, 2-point **(B)**, and 3-point **(C)** effective actions as focal behavior for 2012 (purple) and 2016 (red) tournaments. The behavioral map was represented divided into four quadrants, with each of the conditioned categories as vectors in the axis X/Y and their respective coordinates Z_sum_ prospective (X) and Z_sum_ retrospective (Y). Significate vectors (Radium > 1.96), Zsum values, prospective (pros.) and retrospective (retr.) perspectives, quadrants (Q), radium length (r), and vector angle (θ) are represented. CUT, Cuts; DIA, Direct Attack; PCA, Posterior Counterattack; LIN, linear technique; CIR, Circular technique.

Polar coordinate analysis (Figure 3) showed that, in 2012 Olympic Games, circular techniques (QI, *r* = 2.15) occurred prior to and after one-point actions. To score two points cuts occurred prior to and after scoring (QI, *r* = 5.27) and posterior counterattacks after scoring (QIV, *r* = 2.08). To score three points, linear techniques occurred prior to and after scoring (QI, *r* = 3.04). In 2016 Olympic Games, athletes used direct attacks prior to (QII, *r* = 2.36) one-point actions and cuts after scoring (QIV, *r* = 2.12). Cuts (QII, *r* = 2.12) preceded three-point actions while direct attacks (QIV, *r* = 2.36) occurred after scoring.

## Discussion

This is the first study to follow two medalists through two Olympic Championships and the first that performed both a lag sequential and a polar coordinate analysis to discover the effective patterns in terms of tactics, techniques and the kicking zone associated with the different scores in taekwondo using an observational methodology. As such, it is difficult to compare our results with those of previous studies. The main findings of the present study are that there are specific behaviors used by taekwondo elite athletes to achieve different scores, in accordance with previous studies that consider tactics, techniques, and the kicking zone to be related to score (Matsushigue et al., 2009; Falcó et al., 2012; Falco et al., 2014; Sledweski et al., 2015; Menescardi et al., 2019).

### Effective One-Point Actions

The results showed that both athletes use direct attacks to the chest to score one point (*Z*_0_ = 2.46 and 8.01, respectively). This is in line with previous studies which have reported direct attacks to the chest as the most frequent actions performed during competitions at all competitive levels, whether Olympic, college, or national level tournaments (Matsushigue et al., 2009; Falcó et al., 2012; Sledweski et al., 2015). Further, before scoring, both athletes performed direct attacks (*Z*_−2_ = 2.51, Table 2; *r* = 2.58, Figure 1) which can be interpreted as a way for the athletes to adjust the distance to perform the posterior technique to succeed. After scoring, these two athletes continued with a technique to the head trying to continue scoring (CAB: *Z*_+1_ = 2.46), by adding three or four points depending on the technique performed (World Taekwondo Federation, 2018). This can be achieved due to the fact that when the opponent received a score to the chest, for a short period of time, focused on the area that was not properly blocked (i.e., the chest). It seems that both Jones and Dae-Hoon take this instant opportunity to get points by kicking to the head.

In taekwondo, as in other combat sports, it is important to take into consideration the stimulus-response in order to anticipate opponent's actions and act accordingly to win the bout (Roi and Bianchedi, 2008); such testing is vital to an adequate tactical approach in an adversary sport such as taekwondo. In this sense, Jones used simultaneous counterattacks with circular techniques before scoring while she continued by performing the same pattern (QI, *r* = 2.13 and 3.46, respectively, Figure 2) but to the head after scoring (*Z*_+1_ = 2.10, Table 3). On his side, Dae-Hoon performed direct attacks before (QII, *r* = 2.47) scoring while after scoring he typically did a cut (QIV, *r* = 3.22, Figure 2). These results are in line with previous studies (e.g., Menescardi et al., 2019) that identified that females used cuts or direct attacks with circular techniques to the chest before scoring while after scoring they perform cuts and circular techniques to the head. Also, the same study reported that males perform dodges or anticipations with spinning techniques before scoring while they use simultaneous and anticipation counterattacks with spinning techniques after scoring. It can be anticipated that Jones and Dae-Hoon used their cuts and direct attacks to adjust their distances and test the reaction of the opponent. Taekwondo athletes spend time in well-defined distances from their opponent, which allows to perceive how to better reach the target and score (Falco et al., 2009), highlighting in this case the distance control of both the elite athletes studied. After scoring, they performed a kick to the head (with a circular technique in the case of Jones) that makes the opponent to go back and increase the distance or thereafter cutting the distance (in the case of Dae-Hoon) which requires a new scenario. Those actions showed a specific pattern to finish the sequence.

In both competitions (London 2012 and Rio de Janeiro 2016) both athletes scored one point with techniques to the chest according to the sport's regulations. Before scoring they performed direct attacks, but while in London they used more circular techniques (Menescardi and Estevan, 2017), whereas in Rio de Janeiro the tendency was inverted and they used more linear ones (*Z*_0_ = 2.22, Table 4; Sledweski et al., 2015). After scoring, in London 2012, they used circular techniques, while in Rio 2016, they used cuts, and indirect attacks to the head. This shows the constant evolution of play patterns, not only at different competition level, but also over the years as reported in the sport field (Menescardi et al., 2018).

### Effective Two-Point Actions

To score two points, a spinning technique was used (*Z*_0_ = 4.90, Table 2) in line with previous studies (López-López et al., 2015) and sport's regulations (World Taekwondo Federation, 2012). Before scoring athletes performed cuts (*Z*_−2_ = 4.76; Table 2). Cut an attack can give the opportunity to check how the opponent perform a technique, obtaining the information needed to read the right time and distance to perform a spinning technique, at the same time as the opponent performs her/his attack (i.e., simultaneously).

After scoring athletes performed posterior counterattack (*Z*_+1_ = 4.18) or cuts (*Z*_+2_ = 3.52). Previous studies (Menescardi et al., 2019) have also shown that females tend to perform direct attacks to the head after scoring, which is a perfect combination for a simultaneous or a posterior counterattack as Falco et al. (2014) highlighted. On the other side, the same study reports that males tend to perform simultaneous and anticipatory counterattacks after scoring. Based on that, it seems that athletes adapts their tactics to those situations by performing counterattacks.

Regarding differences between competitions, it should be noted that no effective actions of two points were scored in the 2016 tournament as regulations changed (in 2015) increasing the score from two to three points for spinning actions, highlighting the dynamic nature of this sport and its regulations to make it more popular and attractive to the audience. Therefore, everything related to two point actions can be only attributed to London 2012.

### Effective Three-Point Actions

To score three points, both athletes performed indirect and posterior counterattacks (*Z*_0_ = 2.11 and 2.32, respectively) to the head (*Z*_0_ = 7.68). Before scoring, they performed dodges (*Z*_−2_ = 2.76) while after scoring, they performed direct attacks (QIV, *r* = 2.91, Figure 1), with linear actions (*r* = 2.56), to the chest (*Z*_+1_ = 2.33, Table 2). As highlighted by Falco et al. (2009), it is interesting to observe that before attacking these athletes spent quite some time in well-defined relative distances from each other, as dodges were performed trying to find the optimal distance to attack. It should be noted that a different pattern were obtained for one-point (where they follow up with a technique to the head) than when the athletes obtain a three-point scores to the head (where they follow up with the technique performed to the chest). It can be due to the fact that, after being kicked in the head, athletes tend to raise the arms and at this time, both Jones and Dae-Hoon tried to take profit of this situation by kicking to the chest.

Individual observations showed that before and after scoring Jones primarily used linear techniques (QI, *r* = 3.36, Figure 2), and scored through indirect attacks (*Z*_0_ = 2.74, Table 3). In a recent study, Menescardi et al. (2019) reported that before scoring females performed posterior counterattacks while after that they performed spinning techniques. In this sense, it seems that Jones adapted her tactics to the characteristics of the bout situation and her opponents in an attempt to control them to score (Chiodo et al., 2012; Falco et al., 2014; Menescardi et al., 2015). Here, we can see an evolution of the tactics highlighted by Falco et al. (2014), wherein the best way to react to a posterior counterattack is an indirect attack, while to a spinning kick a linear technique is better. On his side, Dae-Hoon primarily scored by the use of posterior counterattacks (*Z*_0_ = 2.13, Table 4). Before scoring, he preferred the use of indirect attacks (*Z*_−2_ = 2.28) with circular techniques (*r* = 2.06) and cuts (*Z*_−1_ = 1.99). After scoring, he tended to perform more direct attacks and simultaneous counterattacks (*Z*_+1_ = 1.99) with circular techniques (*r* = 2.06). Those behaviors are suitable for the maneuvers reported by Menescardi et al. (2019) wherein, before score, males tend to perform blocs while after, they perform openings.

Regarding the tournaments, in London 2012, dodges (*Z*_−2_ = 2.32) and linear techniques were specially used before scoring (*Z*_−1_ = 2.10; QI, *r* = 3.04) while in Rio de Janeiro 2016, cuts were more predominant (QII, *r* = 2.12). After scoring, indirect attacks (*Z*_+1_ = 2.08) with linear techniques were more used in London 2012, while indirect attacks were used to score in Rio de Janeiro 2016 (*Z*_0_ = 2.07) and direct attacks (QIV, *r* = 2.36) to the chest (*Z*_+1_ = 2.10) were more prevalent after scored. This is in line with Sledweski et al. (2015), who reported that in London 2012 the most effective technique was the linear kick to the head (*naerio chagi*). As suggested previously, differences between tournaments are due to the evolution of taekwondo sparring.

### Effective Four-Point Actions

No significant actions were observed to score points in the game plan/strategy from these two athletes. In this sense, Menescardi et al. (2019) also did not find a significant pattern for 4-point scores for female athletes, while males used simultaneous counterattacks before scoring and indirect attacks before and after scoring to the head with spinning techniques. This does not mean that these athletes do not perform techniques that can score points, but the use of 4-point techniques can be reluctant to fail and therefore, use them in a very specific and punctual situation. The lack of 4-point scores can be interpreted as a more conservative style of the athletes as few spinning actions to the head were performed. Nevertheless, the results of the present study highlight the increased level of tactical behavior (i.e., indirect attacks and posterior counterattacks due to the timing recognition needed to perform them correctly; Falco et al., 2014) as scores increased, suggesting that more elaborate tactics help to achieve the highest score possible.

Regarding the lag sequential and polar coordinate analysis used to analyze the data, as previously mentioned, their complementary potential should be noted (Arias-Pujol and Anguera, 2017; Castañer et al., 2017; Tarragó et al., 2017; Anguera et al., 2018). This is because polar coordinates considerably reduce the amount of data (Morillo et al., 2017), but special relationships can be found with lag sequential analysis in each of the lags analyzed, particularly when the number of actions analyzed are not high as occurred with the follow-up of elite athletes. Consequently, future studies that analyze elite athletes to extract successful (and scarce) patterns should consider combining both techniques to gain a clearer insight into what occurs during competitive situations.

### Limitations and Future Research Lines

As for the limitations of this study, the degree of generalization or the external validity of the results obtained was based on the selected behaviors of only two elite athletes (Olympic athletes). Further studies should consider increasing the number of lags analyzed in order to explain longer sequences to identify the patterns extracted. Additional studies could examine athletes' behavior at different competitive levels (international, college, junior, cadet championships), consider the match outcome (Gómez et al., 2016a,b) and include a greater number of bouts to provide more data to validate the findings of this study.

Independently of the limitations of the present work, studies on taekwondo technical-tactical performance related to scoring that take into account of the multiple factors involved in performance will provide data to help to improve athletes' decision-making in real sparring situations, in addition to allowing coaches and psychologists to train athletes in order to prepare them to act according to the successful patterns extracted. Furthermore, the findings of the present study could be related to the structuring of sport-specific tests, which are able to improve the pertinence of talent identification and selection as encouraged in a previous study (Casolino et al., 2012).

## Conclusions

Athletes have a studied tactical repertoire and use all tactics according to the different types of score and which actions are the most effective. To each scoring option, the athletes use a specific pattern. The pattern observed was that, to score one point, they use direct attacks to the chest. To score two points, they use simultaneous counterattacks with a spinning technique. To score three points, they perform indirect and posterior counterattacks to the head. In addition, before scoring, the athletes prepare the situation to the best suitable instant to the better chance. In that sense, effective one-point actions were preceded by attacks, spinning kicks of two points occurred after cuts while effective three-point actions were preceded by cuts and dodges. After scoring they showed a previously planned performance, either to continue scoring neither to prevent being scored. The athletes performed kicks to the head after one-point actions, two-point actions were followed by posterior counterattacks while three-point actions were followed by direct attacks with linear techniques to the chest. Coaches and trainers should use the described patterns to train athletes according to the successful patterns extracted and avoid the techniques and tactics that follow the effective actions as these patterns were not related to a successful pattern counterpart.

## Data Availability Statement

The datasets generated for this study are available on request to the corresponding author.

## Author Contributions

CM, CF, and AH-M conceived the study, participated in its design and coordination, contributed to video coding, data collection, conducted statistical analyses and contributed to the interpretation of the results, drafted the manuscript, and approved the final manuscript as submitted. CR and VM-S participated in the study design, contributed to the interpretation of the results, reviewed and provided feedback to the manuscript, and approved the final manuscript as submitted. All the authors made substantial contributions to the final manuscript.

### Conflict of Interest

The authors declare that the research was conducted in the absence of any commercial or financial relationships that could be construed as a potential conflict of interest. The handling editor declared a shared affiliation, though no other collaboration, with one of the authors CR at time of review.
