# Supplementary material for: Technical-Tactical Actions Used to Score in Taekwondo: An Analysis of Two Medalists in Two Olympic Championships
Source: Front Psychol. 2019 Dec 10;10:2708. doi: 10.3389/fpsyg.2019.02708 (PMC6914813; doi:10.3389/fpsyg.2019.02708)
Supplement: Supplementary file 6 [file Image_1.pdf]

## Supplemental material

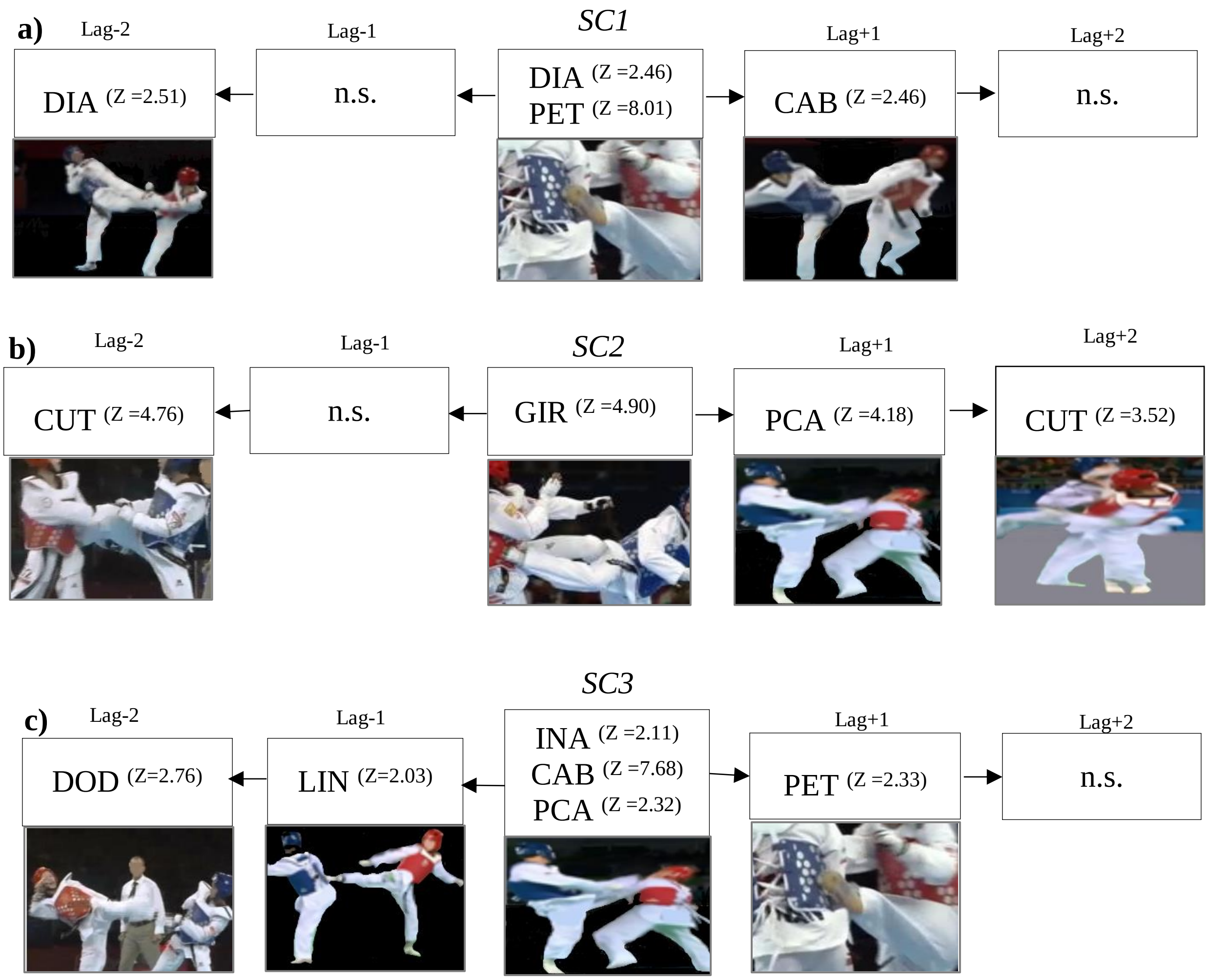


Figure S1. Representation of behavioral patterns of the pooled data extracted from lag sequential analysis. Excitatory relationships, Z values of retrospective lags (-2, -1), focal behavior (lag 0 which corresponds to SC1, SC2 or SC3, respectively in a, b or c) and prospective lags (+1, +2) are represented. n.s. indicates lag with no significant relationships due to athlete's variable behavior. DOD = dodges, CUT = Cuts, OPE = Opening, DIA = Direct Attack, INA = Indirect attack, ACA = Anticipatory Counterattack; SCA = Simultaneous Counterattack; PCA = Posterior Counterattack, LIN= linear technique, CIR = Circular technique; GIR = Spinning technique, PET = Action to the chest protector, CAB = Action to the Head.

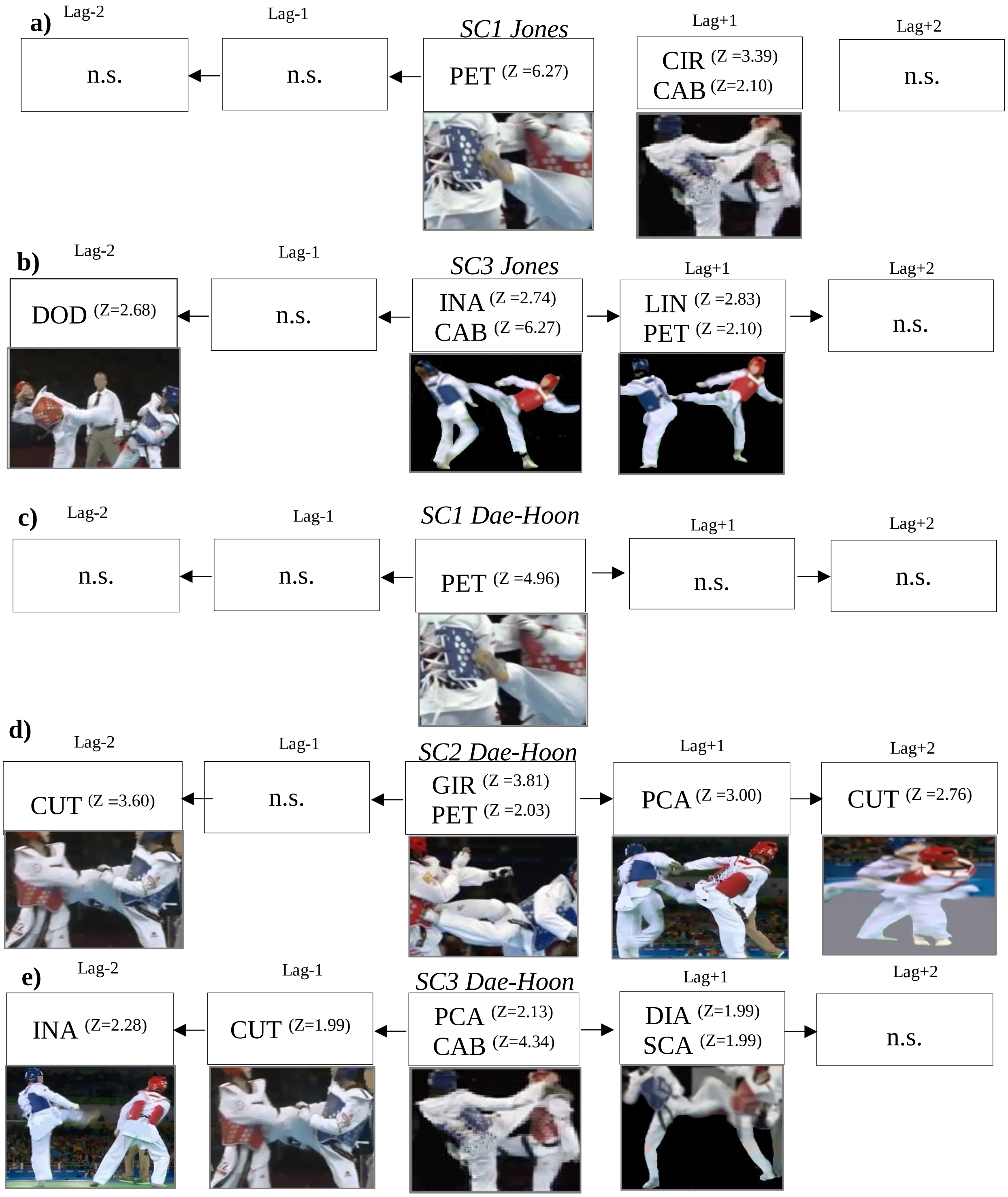


Figure S2. Representation of behavioral patterns of Jones (a and b) and Dae-Hoon (c, d and e) extracted from lag sequential analysis. Excitatory relationships ($Z > 1,96$), Z values of retrospective lags (-2, -1), focal behavior (lag 0 which corresponds to SC1, SC2 or SC3, respectively) and prospective lags (+1, +2) are represented. n.s. indicates lag with no significant relationships due to athlete's variable behavior. CUT = Cuts, DOD = dodges, OPE = Opening, DIA = Direct Attack, INA = Indirect attack, SCA = Simultaneous Counterattack; PCA = Posterior Counterattack, LIN= linear technique, GIR = Spinning technique, PET = Action to the chest protector, CAB = Action to the Head.

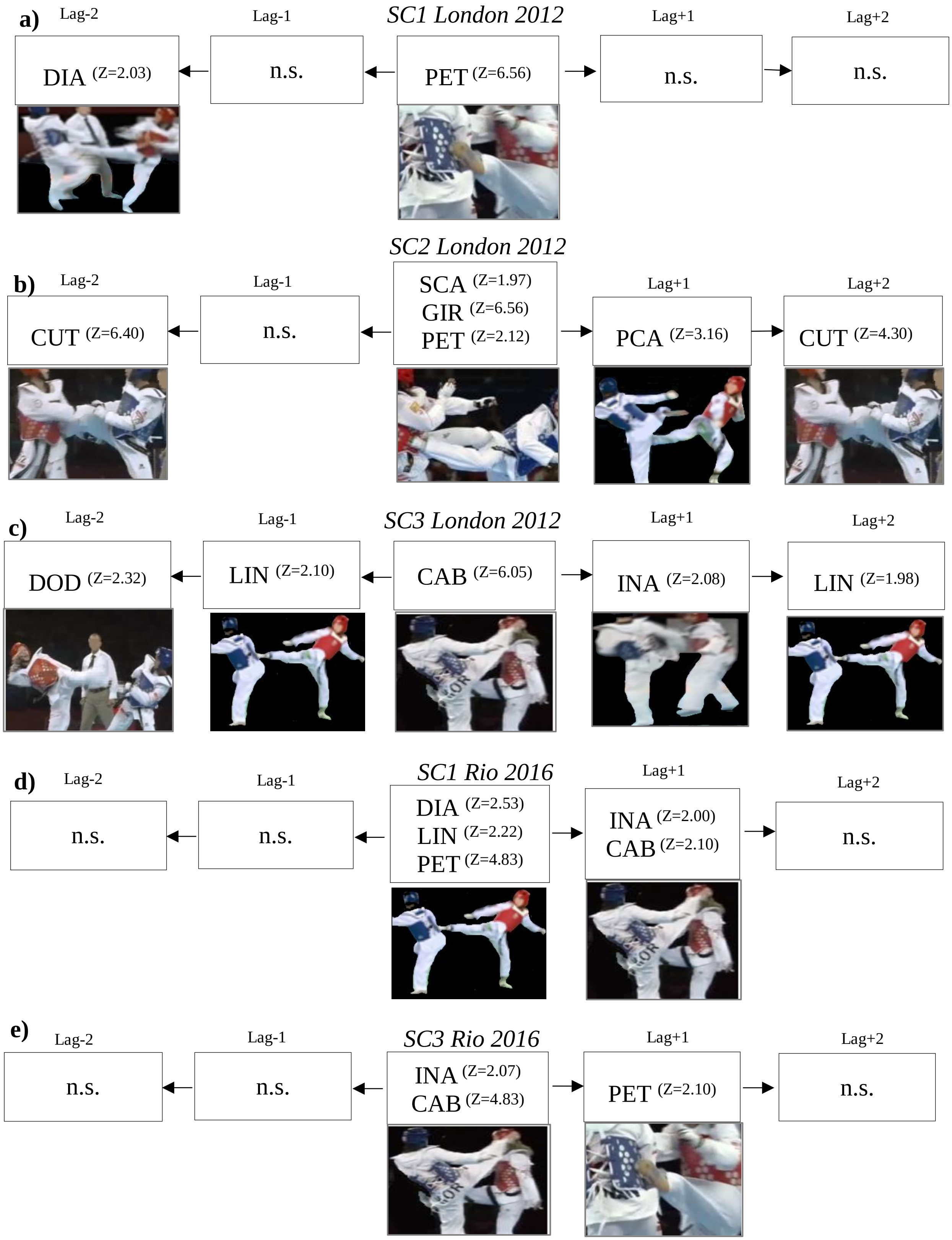


Figure S3. Representation of behavioral patterns of London 2012 (a, b and c) and Rio de Janeiro (d and e) extracted from lag sequential analysis. Excitatory relationships (Z > 1,96), Z values of retrospective lags (-2, -1), focal behavior (lag 0 which corresponds to SC1, SC2 or SC3, respectively) and prospective lags (+1, +2) are represented. n.s. indicates lag with no significant relationships due to athlete's variable behavior. DOD = dodges, CUT = Cuts, DIA = Direct Attack, INA = Indirect attack, SCA = Simultaneous Counterattack; PCA

= Posterior Counterattack, LIN= linear technique, GIR = Spinning technique, PET = Action to the chest protector, CAB = Action to the Head.

## Supplemental media files

Video 1. Sequence performed by Jones to score 1 point by doing direct attack (lag-2), simultaneous counterattack (lag -1), indirect attack with linear technique to the chest (1 point) and circular technique (lag +1) that ended in a cut action prior to embrace the opponent (Figure S2a). Available at https://youtu.be/K5p6bY60TVU

Video 2. Sequence performed by Jones to score 3 points by dodging the opponent (lag-2), simultaneously counterattacking (lag -1), indirectly attacking with linear technique to the head (3 points), kicking with linear technique to the chest (lag +1) prior to embrace the opponent (Figure S2b). Available at https://youtu.be/gjF0eiUsNuA

Video 3. Sequence performed by Dae-Hoon to score 1 point by doing a posterior counterattack (lag-2), a dodge (lag -1), indirect attack (1 point), and direct attacks (lags +1 and +2) (Figure S2c). Available at https://youtu.be/v5ZdKCv6emg

Video 4. Sequence performed by Dae-Hoon to score 2 points by doing a cut action (lag -2), direct attack (lag-1), a simultaneous counterattack with spinning technique (2 points) followed by a dodge (lag +1) and posterior counterattack (Figure S2d). Available at https://youtu.be/1HS3LEq0AcU

Video 5. Sequence performed by Dae-Hoon to score 3 points by doing a cut action (lag -1), posterior counterattack to the head (and to the chest in a combined action to score 3 points) followed by a direct attack (lag +1) (Figure S2e). Available at https://youtu.be/LP8sHDyVQhw
